# Aggregation in Ill-Conditioned Regression Models: A Comparison with Entropy-Based Methods

**DOI:** 10.3390/e27101075

**Published:** 2025-10-16

**Authors:** Ana Helena Tavares, Ana Silva, Tiago Freitas, Maria Costa, Pedro Macedo, Rui A. da Costa

**Affiliations:** 1 Center for Research and Development in Mathematics and Applications (CIDMA), 3810-193 Aveiro, Portugal; lopescosta@ua.pt (M.C.); pmacedo@ua.pt (P.M.); 2 Águeda School of Technology and Management, University of Aveiro, 3750-127 Águeda, Portugal; 3 Department of Mathematics, University of Aveiro, 3810-193 Aveiro, Portugal; 4 Department of Physics, University of Aveiro, 3810-193 Aveiro, Portugalamerico.costa@ua.pt (R.A.d.C.); 5 Institute for Nanostructures, Nanomodelling and Nanofabrication (i3N), 3810-193 Aveiro, Portugal

**Keywords:** big data, collinearity, maximum entropy, normalized entropy, regression modeling

## Abstract

Despite the advances on data analysis methodologies in the last decades, most of the traditional regression methods cannot be directly applied to large-scale data. Although aggregation methods are especially designed to deal with large-scale data, their performance may be strongly reduced in ill-conditioned problems (due to collinearity issues). This work compares the performance of a recent approach based on normalized entropy, a concept from information theory and info-metrics, with bagging and magging, two well-established aggregation methods in the literature, providing valuable insights for applications in regression analysis with large-scale data. While the results reveal a similar performance between methods in terms of prediction accuracy, the approach based on normalized entropy largely outperforms the other methods in terms of precision accuracy, even considering a smaller number of groups and observations per group, which represents an important advantage in inference problems with large-scale data. This work also alerts for the risk of using the OLS estimator, particularly under collinearity scenarios, knowing that data scientists frequently use linear models as a simplified view of the reality in big data analysis, and the OLS estimator is routinely used in practice. Beyond the promising findings of the simulation study, our estimation and aggregation strategies show strong potential for real-world applications in fields such as econometrics, genomics, environmental sciences, and machine learning, where data challenges such as noise and ill-conditioning are persistent.

## 1. Introduction

Large-scale data or big data usually refers to large and complex collections of datasets, which are difficult to process using database management tools or traditional data analysis methodologies. One of the biggest challenges associated with processing big data is retaining relevant statistical information while being computationally efficient. Most large-scale data are also inhomogeneous, meaning they are neither i.i.d. (independent and identically distributed) nor stationary observations from a statistical distribution, as well as noisy, dynamic and inter-related, leading to possible severe ill-conditioning problems.

One way to address the computational burden is through aggregation methods that can be easily implemented in parallel. These consist of the following steps: (i) select *G* groups from the large-scale data (these groups may be overlapping and not include the entirety of the observations); (ii) obtain the vector of estimates, ${\hat{\beta }}_{g}$, using standard techniques (e.g., ordinary least squares, ridge, generalized maximum entropy) for each group *g*, g=1,2,…,G; and, finally, (iii) aggregate the ensemble of vectors of estimates into a single and final vector of estimates, ${\hat{\beta }}_{agg}$.

Costa and Macedo [1] introduced neagging, a new approach for aggregation based on normalized entropy, a concept from information theory and info-metrics [2,3,4,5], and its precision accuracy was compared only with bootstrap aggregating or mean aggregation (bagging) [6] through a simple simulation study. This work substantially improves the study of [1] by presenting results from a large simulation study with 48 different scenarios, where a two-step procedure was adopted: estimation is first performed using OLS or a maximum-entropy-based technique, and the resulting group estimates are then aggregated through bagging, magging (maximin aggregating [7], a recent and powerful aggregation method), or neagging. The purpose of this approach is to enhance the tractability of large-scale data analysis by decomposing the estimation of a potentially massive dataset into the estimation of multiple smaller subsets, which are then recombined into a final aggregated estimate. Precision and prediction accuracies of the combined two-step procedures are compared.

The remaining article is organized as follows: maximum entropy estimation and the aggregation methods discussed in the work are briefly presented in the next section; then follows the simulation study and the discussion of the results; finally, in the last section the most relevant conclusions are provided.

## 2. Methods

In this section are briefly presented two maximum entropy estimators, namely, the generalized maximum entropy (GME) estimator and the weighted generalized maximum entropy (W-GME) estimator, and the three aggregation methods discussed in this work, namely, the bootstrap aggregating or mean aggregation (bagging), the maximin aggregating (magging), and the normalized entropy aggregating (neagging).

### 2.1. Maximum Entropy Estimation

Info-metrics, by generalizing Jaynes’ maximum entropy (ME) principle [2,3]—which itself builds on Shannon’s concept of entropy [8]—relies fundamentally on the notions of information, uncertainty, and entropy. In this framework, information refers to the meaningful content, context, and interpretation of data; uncertainty arises from propositions or outcomes not known with certainty and is represented by probability distributions; and entropy quantifies the expected average information from observations, serving as a measure of uncertainty. The ME principle was taken by [2,3,4] as the basis for transforming the information in the data into a probabilistic distribution that reflects the uncertainty about individual outcomes, and was used by the same authors to develop analytical and empirical methods for recovering the unobservable parameters of the pure linear inverse problem,

(1) y=Xp,

where y is a vector (N×1) of noisy observations, X is a non-invertible matrix (N×K) with N<K, and p is the vector (K×1) of unknown probabilities. From all the probability distributions that satisfy model (1), the ME principle allows us to pick an unambiguous estimate of p by choosing the probability distribution that maximizes Shannon´s entropy,

(2) $$H\left(p\right)=-\sum_{k=1}^{K}p_{k}\ln p_{k}=-p^{\prime }\ln p,$$

subject to the model consistency restrictions, y=Xp, and the additivity restriction, $p^{\prime }1=1$ (note that $p^{\prime }$ is the same as $p^{T}$, representing the transpose of p). Given both these constraints, the ME estimator is formally given by

(3) $$\underset{p}{\mathrm{argmax}}\left\{-p^{\prime }\ln p\right\}.$$

The ME principle provides a tool to make the best prediction using only the available information. The maximization problem in (3) does not have a closed-form solution, which means that the solution must be found with numerical optimization procedures. Using the Lagrange multipliers method (Lagrangian function and first-order optimality conditions), it follows that

(4) $${\hat{p}}_{k}=\frac{\exp(-x_{k}^{\prime }\hat{\lambda })}{\sum_{k=1}^{K}\exp\left(-x_{k}^{\prime }\hat{\lambda }\right)},$$

where $x_{k}$ is a (N×1) vector corresponding to the *k*th column of X and $\hat{\lambda }$ is a (N×1) vector of estimated Lagrange multipliers on the model consistency restrictions. Jaynes’ maximum entropy framework allows us to approach the inverse problem as an inference problem, using optimization to derive a probability distribution that best represents the information in the data and the uncertainty about possible outcomes.

Let us now turn our attention to the linear regression model that is usually represented as

(5) y=Xβ+e,

where, as before, y is the vector (N×1) of noisy observations, X is a known design matrix (N×K) of explanatory variables, β is the vector (K×1) of unknown parameters to be estimated, and e is a (N×1) vector of random disturbances (errors), usually assumed to have a conditional expected value of zero and representing spherical disturbances, i.e., E[e|X]=0 and $E\left[{\mathrm{ee}}^{\prime }|X\right]=\sigma^{2}I$, where I is the (N×N) identity matrix and $\sigma^{2}$ is the error variance.

The ordinary least squares (OLS) estimator of β in the model (5), given by

(6) $$\hat{\beta }:={\left(X^{\prime }X\right)}^{-1}X^{\prime }y,$$

is probably the most known and widely used estimator in linear regression. However, in the presence of collinearity (also known as ill-conditioning, referring to a near-linear relationship between two or more regressors), and with its increasing severity, the vector $\hat{\beta }$ obtained by the OLS estimator can be expected to be farther from the vector β; e.g., [9,10,11].

The ME principle can be extended to estimate the linear regression model in this context, and to deal with ill-posed problems, where collinearity is included, A. Golan and coauthors [4] proposed a reparameterization of the model in (5) as

(7) y=XZp+Vw,

where

(8) $$\beta =\mathrm{Zp}=\left[\begin{array}{cccc} z_{1}^{\prime } & 0 & \cdots & 0\\ 0 & z_{2}^{\prime } & \cdots & 0\\ \vdots & \vdots & \ddots & \vdots \\ 0 & 0 & \cdots & z_{K}^{\prime } \end{array}\right]\left[\begin{array}{c} p_{1}\\ p_{2}\\ \vdots \\ p_{K} \end{array}\right],$$

with Z a (K×KM) matrix of support spaces (closed and bounded intervals in which each parameter is restricted to belong) and p a (KM×1) vector of unknown probabilities to be estimated, and

(9) $$e=\mathrm{Vw}=\left[\begin{array}{cccc} v_{1}^{\prime } & 0 & \cdots & 0\\ 0 & v_{2}^{\prime } & \cdots & 0\\ \vdots & \vdots & \ddots & \vdots \\ 0 & 0 & \cdots & v_{N}^{\prime } \end{array}\right]\left[\begin{array}{c} w_{1}\\ w_{2}\\ \vdots \\ w_{N} \end{array}\right],$$

with V a (N×NJ) matrix of support spaces (closed and bounded intervals in which each error is restricted to belong) and w a (NJ×1) vector of unknown probabilities to be estimated. In this context, each $\beta_{k}$, k=1,2,…,K, and each $e_{n}$, n=1,2,…,N, are viewed as expected values of discrete random variables, with M≥2 and J≥2 as possible outcomes, within the lower and upper bounds of the corresponding support spaces. Thus, considering the linear regression model specified in (5) and assuming independence between p and w (additivity property of Shannon entropy), the generalized maximum entropy (GME) estimator is given by

(10) $$\underset{p,w}{\mathrm{argmax}}\left\{-p^{\prime }\ln p-w^{\prime }\ln w\right\},$$

subject to the model constraints

(11) y=XZp+Vw,

and the additivity constraints for p and w, respectively,

(12) $$\begin{array}{c} 1_{K}=\left(I_{K}⊗1_{M}^{\prime }\right)p,\\ 1_{N}=\left(I_{N}⊗1_{J}^{\prime }\right)w, \end{array}$$

where ⊗ represents the Kronecker product, 1 is a column vector of ones with the specified dimension, and I is an identity matrix with the specified dimension. It is important to note that while the ME estimator was originally designed for pure linear inverse problems (with N<K), the GME estimator broadens the scope of the method and remains valid regardless of the relative sizes of *N* and *K*, thereby encompassing underdetermined, overdetermined, and exactly identified problems. Using numerical optimization techniques, the GME estimator generates the optimal probability vectors $\hat{p}$ and $\hat{w}$ that are used to obtain point estimates of the parameters and the errors through the reparameterizations (8) and (9), respectively. For example, analogously to the solution of the ME estimator in (4), the formal solution of the GME estimator for p is given by

(13) $${\hat{p}}_{km}=\frac{\exp\left(-z_{km}x_{k}^{\prime }\hat{\lambda }\right)}{\sum_{m=1}^{M}\exp\left(-z_{km}x_{k}^{\prime }\hat{\lambda }\right)},$$

where $x_{k}$ is a (N×1) vector corresponding to the *k*th column of X and $\hat{\lambda }$ is a (N×1) vector of estimated Lagrange multipliers on the model constraints (11). Additional details can be found in [4] (pp. 90–93).

Regarding the specification of the support spaces in Z, for the parameters of the model, the bounds are usually obtained based on theoretical constraints or information from previous research. Nevertheless, wide symmetric supports about zero, usually with five equally spaced points (M=5) between the lower and upper bounds, should be used whenever there is no a priori information available. The bounds for the support spaces in V, for the errors of the model, are usually obtained by the three-sigma rule [12], considering the standard deviation of the noisy observations (observed dependent/response variable) rounded up to the nearest integer, usually with three points (J=3) symmetric about zero. Additional details about support spaces can be found in [4,5,13], and an illustrative example of the matrix structure of the GME estimator is given in Appendix A, also discussing the reason to consider two different scenarios for supports in Section 3.

X. Wu [14] proposed the weighted generalized maximum entropy estimator with a data-driven weight, where the objective function in (10) is updated as

(14) $$\underset{p,w}{\mathrm{argmax}}\left\{-\left(1-\gamma \right)p^{\prime }\ln p-\gamma w^{\prime }\ln w\right\},$$

where γ∈(0,1) defines the weights assigned to each entropy component in the objective function, and it is selected by some kind of cross-validation through the minimization of a given loss function (e.g., the minimization of the sum of the squared prediction errors). Under different simulated scenarios, the author illustrates that the W-GME estimator provides superior performance than the GME estimator [14].

### 2.2. Aggregation Methods

The bagging method [6] simply averages, with uniform weights, over the ensemble of estimates from the groups, and the global estimate is given by

(15) $${\hat{\beta }}_{agg}:=\sum_{g=1}^{G}w_{g}{\hat{\beta }}_{g},$$

where $w_{g}=\frac{1}{G}$ for all g=1,2,…,G. It is a simple method where the estimates are obtained from bootstrap (random with replacement) samples. However, it is not considered in this work the case of a single learning set and the need to take repeated bootstrap samples from it as in [6].

The magging method [7] builds an aggregate estimator as a convex combination of group-specific estimators, ${\hat{\beta }}_{g}$. The weights $w_{g}$ are chosen not to minimize prediction error in an average sense like in bagging, but to minimize the $l_{2}$-norm of the fitted values across groups, which can be interpreted as maximizing the “worst-case” explained variance across the different groups. This ensures that the final aggregate model captures the effects that are consistently present in all groups, rather than the effects that are strong in some groups but absent in others. Explicitly, the global estimate is given by

(16) $${\hat{\beta }}_{agg}:=\sum_{g=1}^{G}w_{g}{\hat{\beta }}_{g},$$

where

(17) $$w:=\underset{w\in W}{\mathrm{argmin}}∥\sum_{g=1}^{G}w_{g}{\hat{y}}_{g}∥,$$

with the constraint $W=\{w:\underset{g}{\min}\;w_{g}\geq 0\;\mathrm{and}\;\sum_{g=1}^{G}w_{g}=1\}$. As mentioned above, the weights are chosen as a convex combination to minimize the $l_{2}$-norm of the vector of fitted values, ${\hat{y}}_{g}=X{\hat{\beta }}_{g}$, and this method can be easily implemented with quadratic programming. If the solution is not unique, it is considered the solution with lowest $l_{2}$-norm of the weight vector among all solutions. The idea behind magging is that if an effect is common across all groups, then it cannot be “averaged away’’ by searching for a specific combination of the weights [7].

The neagging method, the approach for aggregation based on normalized entropy [1,15], consists of weighting the estimate obtained from the GME estimator [4,5,16] for each individual group according to the amount of information in that group, because if it is true that groups obtained from random sampling carry information about the whole dataset, they are of course not equally informative. To measure the information content in a system with *K* states, the normalized entropy, $S(\hat{p})$ can be used, as defined by

(18) $$S\left(\hat{p}\right):=\frac{-\sum_{k}\hat{p_{k}}\ln\hat{p_{k}}}{\ln K},$$

where $S\left(\hat{p}\right)\in \left[0,1\right]$, and ln(K) represents maximum uncertainty, which is the entropy level of a uniform distribution with *K* outcomes [4]. The normalized entropy aggregation scheme, neagging, is based on identifying the information content of a given group, *g*, through the calculation of the normalized entropy associated with the estimate of that group,

(19) $$S{\left(\hat{p}\right)}_{g}:=\frac{-{\hat{p}}^{\prime }\ln\hat{p}}{K\ln M},$$

where *M* is number of support points in the specific group *g*, and the denominator, KlnM, represents maximum entropy, necessary for normalization. The weights in the aggregation scheme are calculated such that $w_{g}\propto 1-S{\left(\hat{p}\right)}_{g}$ and $\sum_{g=1}^{G}w_{g}=1$. The global estimate is then given by

(20) $${\hat{\beta }}_{agg}:=\sum_{g=1}^{G}w_{g}{\hat{\beta }}_{g}.$$

The resulting final estimates will be a weighted average of the ensemble of vectors with estimates based on the information content of each group, and this method is almost as simple as bagging, with the weights expected to be non-uniform as in magging.

## 3. Simulation Study

The simulation setup is designed to emulate high-dimensional regression problems with strong collinearity and different error distributions. Such settings are commonly found in econometric modeling, genomics, environmental studies, and machine learning applications. The synthetic datasets reflect these structures in a controlled way, allowing for a focused evaluation of different methods (estimation and aggregation) under ill-conditioning and data heterogeneity scenarios. All the code is developed in MATLAB (R2019b version) [17] by the authors.

The general context of the simulation is as follows: the two collinearity scenarios are intended to illustrate models with low and high collinearity found in practice; the vector of parameters illustrates scenarios “close” to zero, positive and negative, very common in practice; the different error distributions reflect variations in noise; the size of the models is still computationally feasible for the design of the simulations and allows for the possibility of sampling; the two parameter supports for GME and W-GME reflect reduced and high information by the user regarding the model parameters; the supports for the errors, as well as the number of points in the supports, are the usual ones in the literature. Finally, the number of groups and the number of observations per group try to reflect the purpose of aggregation (obtaining accurate information with minimum computational effort and observing a small part of the population under study).

### 3.1. Simulation Settings

A linear regression model is considered, with a number of observations of 30,000 and a number of explanatory variables of 10 (*N* = 30,000 and K=10). Two X (30,000 × 10) matrices of explanatory variables are simulated, corresponding to matrices with two different condition numbers, representing two distinct collinearity scenarios: cond(X) = 10, for cases of near absence of collinearity; and cond(X) = 20,000, for cases where high collinearity is present in the data. The X matrices were initially constructed using standard normal distributions, and then the singular value decomposition was used to algebraically adjust the matrices to the desired condition numbers. The MATLAB functions available imposed a restriction on the size of the matrices that could be used, and the values N×K = 30,000 × 10 represented the maximum feasible under these conditions. Anyway, the size of the original dataset in the simulation study (whether thousands or millions) is not relevant, as we will always work on estimating parameters in models with small datasets extracted by sampling. The simulation framework assumes a linear relationship between explanatory variables and the dependent variable, with errors following known distributions. These assumptions were chosen deliberately in order to provide a controlled environment where the effects of ill-conditioning, choice of the estimators, and aggregation methods could be systematically isolated and evaluated. However, it is also important to note that data scientists dealing with huge amounts of data frequently use linear models as a simplified view of the reality and these are actually very satisfactory models quite often.

The number of parameters in the regression model will correspond to the number of explanatory variables and, consequently, the parameter vector β will be a (10×1) vector, defined as β=[2,1,−3,5,−5,3,4,−2,−1,−4], not containing the constant term. Furthermore, three distinct types of error are considered for the given datasets: errors modeled by a normal distribution with zero mean and unit standard deviation, e∼N(0,1), to represent scenarios with relatively low noise; errors based on a t-Student distribution with three degrees of freedom, e∼t(3), to represent scenarios with significant but moderate noise; and errors following a Cauchy distribution with location parameter zero and scale parameter two, e∼C(0,2), to represent scenarios with high noise. In this way, three distinct vectors e (30,000 ×1) of random perturbations are obtained, resulting in six vectors y (30,000 ×1) of noisy observations, combining the two different condition numbers with the three vectors of random perturbations, to obtain a wide variety of noisy observation vectors with different characteristics.

The reparameterizations imposed by the GME and W-GME estimators are carried out by defining the matrix V, which contains the supports of the errors, with symmetric supports centered at zero, using the 3-sigma rule, where sigma is approximated by the empirical standard deviation of the noisy observations, and the support is always the same for each unknown perturbation. Consequently, the support vectors of the errors will take the form $v_{n}=\left[-3\hat{\sigma },0,3\hat{\sigma }\right]$ for all n=1,…, 30,000, being equally spaced, with J=3 points used to form these supports. Additionally, two distinct supports for the parameters are considered, both symmetric and centered at zero, so these vectors will also be equally spaced, considering M=5 points to form these supports (the same for each of the unknown components of the parameter vector of the model). Thus, two distinct matrices Z are defined, containing the supports for the parameters: one with $z_{k}=\left[-10,10\right]$ for all k=1,…,10, to reflect scenarios where there is some prior knowledge about the range in which the parameters may be found, allowing for a reduction in the amplitude of the parameter supports; and another with $z_{k}=\left[-200,200\right]$ for all k=1,…,10, to reflect scenarios where the available prior knowledge is insufficient. As a clarification remark, the support vectors are actually given by $z_{k}=\left[-10,-5,0,5,10\right]$ and $z_{k}=\left[-200,-100,0,100,200\right]$, but, to simplify notation, they will be mentioned henceforth as $z_{k}=\left[-10,10\right]$ and $z_{k}=\left[-200,200\right]$. The objective is to understand (albeit preliminarily, as this is not the main aim of the work) how the amplitude of the parameter supports affects the results of the GME and W-GME estimators.

Completing with the necessary specifications regarding the aggregation methods, random sampling with replacement was performed considering two values for the number of groups, namely, G=10 and G=20, with the aim of analyzing whether increasing the number of groups in each aggregation method causes significant differences in the results obtained, and two values for the number of observations per group, Obs =50 and Obs =100, to assess whether the increase in observations in each group, that is, the increase in information available in each group, produces relevant changes in the performance of each of the aggregation methods.

In summary, a total of 48 scenarios will be analyzed, referring to the combination of the two matrices of explanatory variables (two condition numbers) and the three error vectors (three error distributions), generating a total of six different datasets, associated with the two supports for the parameters, the two values for the number of groups in the aggregation methods, and the two values for the number of observations per group, forming a total of eight variants of structures. Additionally, a key point of this simulation study will also be to study the variance of the results, as there is a random sampling process involved, to understand whether the results obtained are directly associated with the aggregation method used or if it is a consequence of a less informative sampling process. For this purpose, Monte Carlo experiments with 10 replicas for each scenario are conducted, with the objective of exploring the sampling behavior of the situations under study.

Once the datasets are obtained, the model coefficients are estimated using the previously mentioned aggregation techniques, bagging, magging, and neagging, varying the estimators used in each aggregation procedure, namely, the OLS, GME, and W-GME estimators. For each of the 48 Monte Carlo experiments related to the scenarios under analysis, the regression coefficient estimates of each considered aggregation method are calculated in each of the 10 replicas, using the different estimators discussed. These estimates will be defined by ${\hat{\beta }}_{aggr}$, expressing the regression coefficients estimated after aggregation. The final regression coefficient estimates, that is, those obtained after conducting the Monte Carlo experiment, relative to each of the aggregation procedures, using the different estimators considered in this study, are calculated by averaging the 10 ${\hat{\beta }}_{aggr}$ values obtained from each replica, being referred to as ${\hat{\beta }}_{\bar{aggr}}$, expressing the final estimated regression coefficients after aggregation (mean of the replicas). Henceforth, when presenting the results of this study, the general abbreviation ‘aggr’ will be replaced by the following abbreviations: bOLS to represent the bagging aggregation procedure with OLS coefficient estimates; bGME to represent the bagging procedure with GME estimates; bW-GME to represent the bagging procedure with W-GME estimates; mOLS to represent the magging procedure with OLS estimates; mGME to represent the magging procedure with GME estimates; mW-GME to represent the magging procedure with W-GME estimates; nGME to represent the neagging procedure with GME estimates; and nW-GME to represent the neagging procedure with W-GME estimates.

### 3.2. Evaluation Metrics Based on Prediction and Precision Errors

For each of the Monte Carlo experiments related to the scenarios under analysis, the prediction and precision errors associated with each of the ${\hat{\beta }}_{aggr}$ obtained are calculated for all 10 replicas. The prediction error is derived from the Euclidean norm of the difference between the vector of predicted observations using the model (multiplication of the matrix of explanatory variables by the estimated regression coefficients after aggregation) and the simulated vector of noisy observations, that is, $||X{\hat{\beta }}_{aggr}-y\left|\right|$. The precision error is calculated through the Euclidean norm of the difference between the vector of estimated regression coefficients after aggregation and the original parameter vector considered in the simulation, that is, $\left|\right|{\hat{\beta }}_{aggr}-\beta \left|\right|$. These two complementary metrics are used to assess the effectiveness of each aggregation strategy: precision error (a standard metric in regression analysis under simulation; reflects the overall stability of the parameter estimates across replications) and prediction error (a standard metric in regression analysis; reflects the practical predictive accuracy of the estimated model).

The prediction error 1 and precision error 1 are obtained by averaging the 10 prediction and precision errors obtained in each replica, thus obtaining $\left|\right|\hat{y}-y\left|\right|$ and $\left|\right|\hat{\beta }-\beta \left|\right|$, respectively, and the prediction error 2 and precision error 2 are determined by implementing the formulas for prediction and precision errors, but using the final estimated regression coefficients after aggregation (mean of the replicas), ${\hat{\beta }}_{\bar{aggr}}$, obtaining the values $\left|\right|\hat{y}-y{\left|\right|}^{*}$ and $\left|\right|\hat{\beta }-\beta {\left|\right|}^{*}$, respectively.

The variances of the results are calculated using the usual variance formula, that is, the mean of the sum of the squares of the differences between each element and its mean. Therefore, the variance of ${\hat{\beta }}_{\bar{\mathrm{aggr}}}$, the variance of the prediction error, and the variance of the precision error are

(21) $$\begin{array}{cc} s_{{\hat{\beta }}_{\bar{aggr}}}^{2} & =\frac{1}{R}\sum_{i=1}^{R}{\left({\hat{\beta }}_{aggr_{i}}-{\hat{\beta }}_{\bar{aggr}}\right)}^{2}, \end{array}$$

(22) $$\begin{array}{cc} s_{\left|\right|\hat{y}-y\left|\right|}^{2} & =\frac{1}{R}\sum_{i=1}^{R}(||X{\hat{\beta }}_{aggr}-{y||}_{i}-\left|\right|\hat{y}-{y||)}^{2}, \end{array}$$

(23) $$\begin{array}{cc} s_{\left|\right|\hat{\beta }-\beta \left|\right|}^{2} & =\frac{1}{R}\sum_{i=1}^{R}\left(|\right|{\hat{\beta }}_{aggr}-{\beta ||}_{i}-\left|\right|\hat{\beta }-{\beta ||)}^{2}, \end{array}$$

respectively, with *R* corresponding to the number of replicas, 10. While formal hypothesis testing is not applied in this study, the use of replicas per scenario, variance estimates, and boxplot visualizations offer a preliminary assessment of the stability and dispersion of results across different techniques (estimation and aggregation).

## 4. Results and Discussion

The results presented in this work aim to highlight the general trends of this simulation study. To better understand and analyze the results obtained, summary tables of precision errors 1 and 2 for all the previously mentioned scenarios are presented in Table 1, Table 2 and Table 3. Data visualization was carried out using MATLAB for the boxplots and R (ggplot2 package) for the heatmaps.

Additionally, comparative graphs of precision errors between the different methods are presented, when the number of groups and the number of observations per group are modified, in order to evaluate the impact of these factors on the studied aggregation methods, with the respective estimators considered. Due to the extent of this simulation study, given the high number of presented scenarios and subsequent vectors of estimates and variances obtained (eight vectors per scenario, relative to each of the aggregation procedures with the different estimators considered), only the results of certain scenarios are highlighted. These scenarios aim to establish a representative set of the total scenarios considered in the study. The remaining are available upon request to the authors.

The values of the prediction errors 1 and 2 resulting from these selected scenarios, and the variances associated with these results, are found in Table 4, Table 5 and Table 6. Note that values presented as 0.00 do not represent a null value, but, rather, a value less than 0.01. Additionally, boxplots of the regression coefficient estimates and precision error for these scenarios are presented for a graphical analysis (among other aspects) of the variances relative to these results.

Analyzing the precision errors obtained in the scenarios e∼N(0,1), Table 1, in situations of low collinearity (cond(X) = 10), it is observed that $\left|\right|\hat{\beta }-\beta \left|\right|$ is generally lower when one of the estimators based on the ME principle is applied. Although the OLS estimator usually performs well in data with low collinearity, the estimates obtained by procedures using the GME and W-GME estimators stand out compared to the estimates obtained by the bOLS and mOLS procedures. The bGME, bW-GME, mGME, and mW-GME are the methods that distinguish themselves by their good performance. However, the performance of the bagging method with the GME and W-GME estimators tends to worsen with the increase in the width of the coefficient supports, highlighting the better performance of magging with the use of these estimators. Additionally, the performance of mW-GME generally surpasses that of mGME in scenarios where $z_{k}=\left[-200,200\right]$. The nGME also presents one of the best performances, but only in the situation where the support for the parameters is narrower ($z_{k}=\left[-10,10\right]$).

Now, analyzing a scenario with high collinearity (cond(X) = 20,000), a significant increase in $\left|\right|\hat{\beta }-\beta \left|\right|$ is observed in aggregation methods using the OLS estimator, while the methodologies that stood out previously in the scenario with low collinearity continue to behave similarly. This indicates that the presence of collinearity does not seem to affect the results provided by the previously mentioned aggregation methods, namely, those using estimators based on the ME principle. As the performance of the OLS estimator is generally affected by the degree of collinearity in the data, and the GME estimator (and, by extension, the W-GME estimator) is suitable for handling data affected by collinearity, these results are not unexpected. Again, the magging aggregation method with the GME and W-GME estimators stands out for its performance, and the behaviors observed for low collinearity scenarios are also observed in these cases. However, with the increase in G and Obs, the performance of bGME and bW-GME improves once more.

Note that, when evaluating the quality of the obtained estimates (precision error 2), the scenarios with a wider range of parameter supports, a larger number of groups, and a greater number of observations exhibit the most suitable estimates for the regression coefficients of the presented problem, with the use of bGME, bW-GME, and nGME, regardless of the degree of collinearity present (but more visibly with higher collinearity).

When e∼t(3), the performance of bGME and bW-GME decreases compared to the previous case. From Table 2, it can be seen that the methods that consistently present the lowest $\left|\right|\hat{\beta }-\beta \left|\right|$ are mGME and mW-GME. The bagging and neagging aggregation methods generally show a decrease in their performance compared to the previous table, suggesting that these methods are more affected by the presence of larger perturbations in the data. The effects caused by collinearity and the existence of more adverse perturbations are noticeable in the aggregation methods that apply the OLS estimator.

Finally, analyzing the case where e∼C(0,2), Table 3, the conclusions are similar to the case e∼t(3). The bagging and neagging aggregation methods show poor performance, especially when using the OLS estimator, and the magging aggregation method is recognized for its performance when using the GME and W-GME estimators. However, mW-GME reveals a predominantly dominant behavior in terms of precision error compared to mGME (with similar performance or insignificant differences in cases where it does not stand out), particularly in scenarios with $z_{k}=\left[-200,200\right]$.

Subsequently, it is necessary to analyze to what extent the increase in the number of groups (G) and observations per group (Obs) can affect the performance of the various aggregation methods using the considered estimators. Comparing the same support, $z_{k}=\left[-10,10\right]$, with cond(X) = 10 and cond(X) = 20,000, it is observed (illustrative graphical representions highlighting this observation can be requested from the authors) that bOLS demonstrates worse performance when Obs is smaller, and $\left|\right|\hat{\beta }-\beta \left|\right|$ is lower when this number increases. The increase in G seems to improve the performance of bOLS, except in the case e∼C(0,2), which shows an atypical effect on the precision error of bOLS, possibly due to the pronounced presence of noise.

These results are expected due to sampling and inferential statistical theory. The mOLS method seems to follow a similar behavior, where both the increase in G and the increase in Obs lead to better performance, even in cases where e∼C(0,2).

However, the estimators based on the ME principle do not appear to follow this behavior, remaining approximately constant with the increase in G and the addition of more observations per group, being more unequivocal in the bagging and magging aggregation methods. The invariability of $\left|\right|\hat{\beta }-\beta \left|\right|$ values concerning the increase in the number of groups/observations is a significant advantage in using the bagging and magging methods that use the GME and W-GME estimators. As there is no need for a larger number of groups or larger datasets to obtain suitable estimates for the problem in terms of precision error, it is possible to reduce the computational burden of implementing these aggregation procedures.

In summary, as observed in Table 1, Table 2 and Table 3, the best precision error results are obtained through bGME, bW-GME, mGME, and mW-GME in scenarios of errors modeled by a normal distribution, and by mGME and mW-GME in scenarios modeled by a t-Student or Cauchy distribution.

Analyzing the same support $z_{k}=\left[-200,200\right]$, with cond(X) = 10 and cond(X) = 20,000, the conclusions for the aggregation methods employing the OLS estimator remain substantially the same as before (illustrative graphical representations highlighting this observation can be requested from the authors) for all considered error distributions. However, with the increase in the parameter supports’ range (and despite scenarios where e∼N(0,1), in which $\left|\right|\hat{\beta }-\beta \left|\right|$ remains approximately constant for bGME, bW-GME, mGME, and mW-GME), when e∼t(3) and e∼C(0,2), all aggregation procedures with estimators based on the ME principle show a decrease in performance as Obs increases (contrary to the results obtained for the narrower support). Except for the neagging method in the case of e∼C(0,2), this decrease appears to be more pronounced with the increase in the number of observations and remains unchanged with the increase in the number of groups. In the case of the neagging method, this effect might indicate that aggregating estimates with the knowledge of the proportion of the information content of various groups is more advantageous compared to aggregating with the proportion of the information content of larger groups, as groups with many observations do not necessarily imply a higher state of knowledge or a lower state of uncertainty.

Lastly, considering the comparison of situations with the same condition number, and naturally excluding methods that used the OLS estimator, it can be said that the increase in the parameter supports’ range, from $z_{k}=\left[-10,10\right]$ to $z_{k}=\left[-200,200\right]$, caused a significant decrease in the performance of all aggregation procedures, except for mGME and mW-GME (excluding cases with lower G and higher Obs). This analysis is in agreement with the discussion of Table 1, Table 2 and Table 3.

One of the most characteristic aspects of this simulation study was the similar performance of prediction errors for all analyzed scenarios, with differences only occurring with changes in the distribution of random disturbances. The most pronounced difference in the values of $\left|\right|\hat{y}-y\left|\right|$ was associated with bOLS, which exhibits the highest prediction error value and associated variance. However, $\left|\right|\hat{y}-y\left|\right|$ is similar for all other aggregation methodologies and their estimators used, even when changing the level of collinearity present in the explanatory variables, the supports of the unknown parameters, and the number of groups and observations per group. The only exception is in the neagging aggregation methodologies when the supports’ range is broader, producing higher prediction errors and greater associated variance, except when e∼C(0,2). However, the magging aggregation method consistently shows low prediction error and associated variance across all analyzed scenarios.

**Figure 1:**
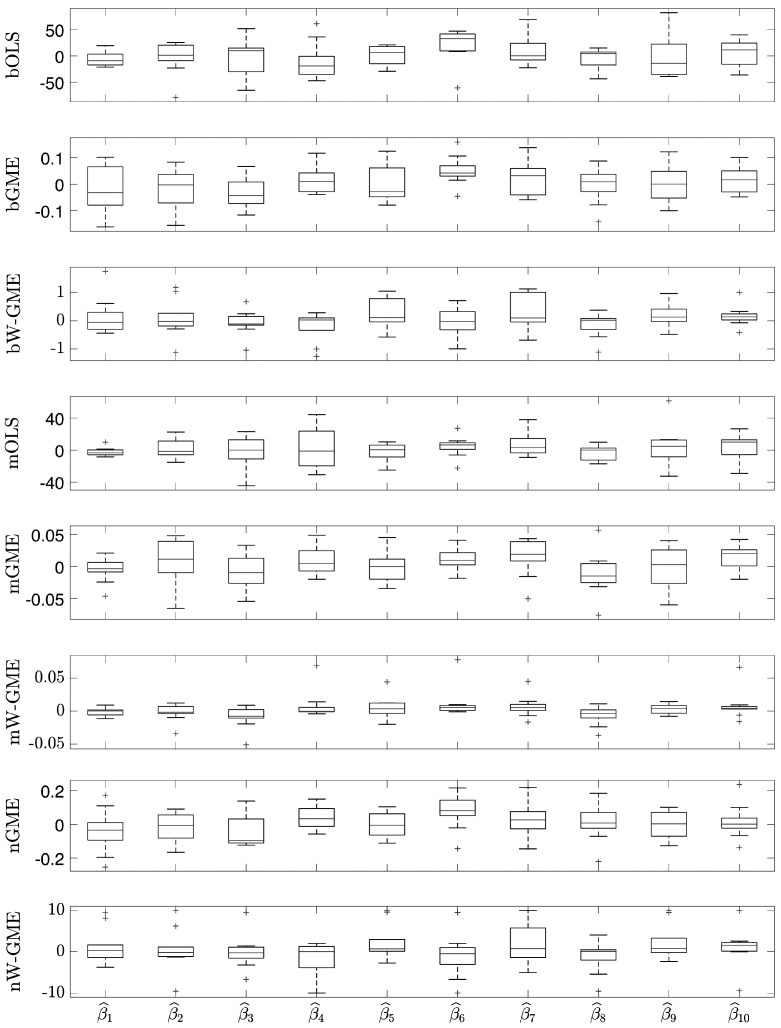
Box plots of the estimates of the 10 linear regression coefficients. Scenario: e∼N(0,1), cond(X) = 10, $z_{k}=\left[-10,10\right]$, G = 10, Obs = 50. Note the different scales inside the figure.

**Figure 2:**
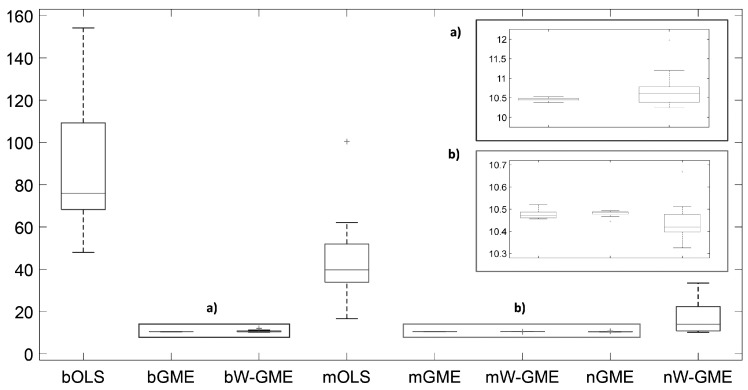
Box plots of the precision errors. Scenario: e∼N(0,1), cond(X) = 10, $z_{k}=\left[-10,10\right]$, G = 10, Obs = 50. (**a**,**b**) are zoomed-in images of the highlighted box plots.

In this initial scenario, the estimates provided by bGME, bW-GME, mGME, mW-GME, and nGME in Table 4 show a high level of shrinkage towards the center of their supports and reduced variance in the results—the GME estimator can be considered a shrinkage estimator. As the support limits are narrow, they exert significant pressure on the estimates, as decreasing the support limits diminishes the impact of the data and increases the impact of the supports. Despite this, due to the prior information provided to the method, they yield better results than the conventional OLS estimator in the context of aggregation. The analysis of the boxplots associated with these estimates shows very little variation, indicating that these procedures are more stable than the other procedures analyzed, as evidenced in Figure 1. Additionally, this behavior is identified in Figure 2, as the widths of the box plot boxes for the precision errors of these procedures are much smaller than those of other methods, indicating reduced variation in the results (please note the different scales inside some figures and between figures throughout this work).

Given this, it can be said that the bagging and magging aggregation methods exhibit more stable estimates for this scenario, but only when applied with the GME and W-GME estimators, as well as the neagging method when applied with the GME.

**Figure 3:**
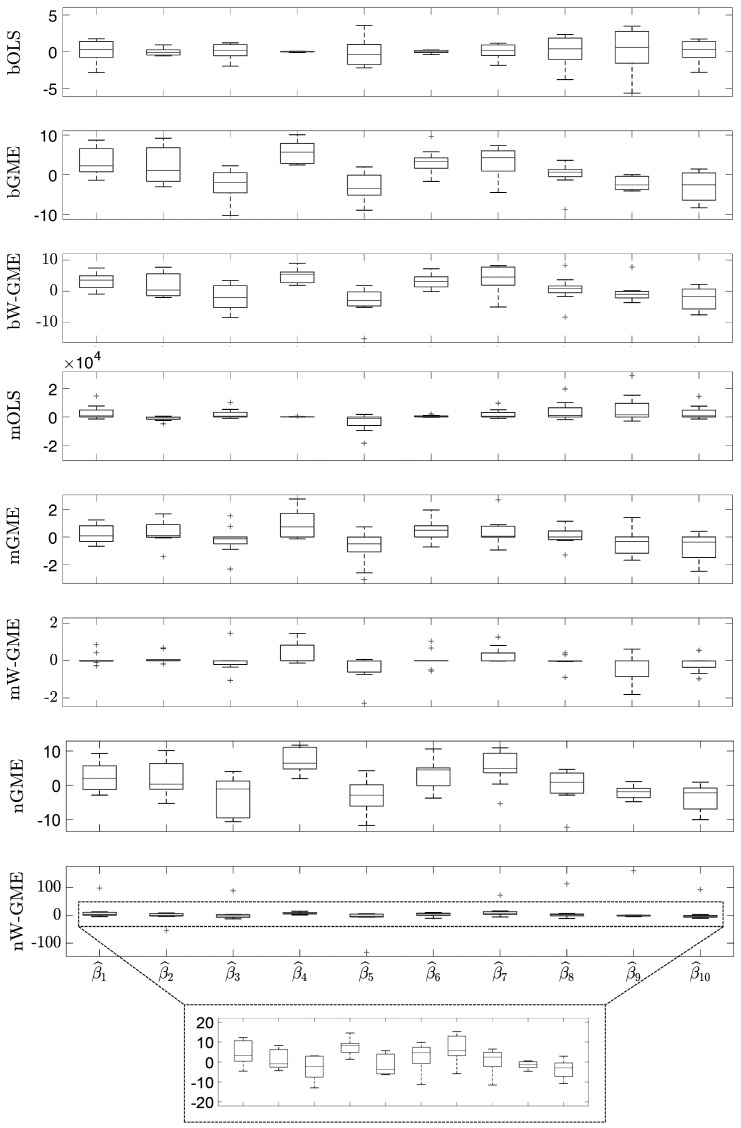
Box plots of the estimates of the 10 linear regression coefficients. Scenario: e∼N(0,1), cond(X) = 20,000, $z_{k}=\left[-200,200\right]$, G = 20, Obs = 100. Note the different scales inside the figure.

**Figure 4:**
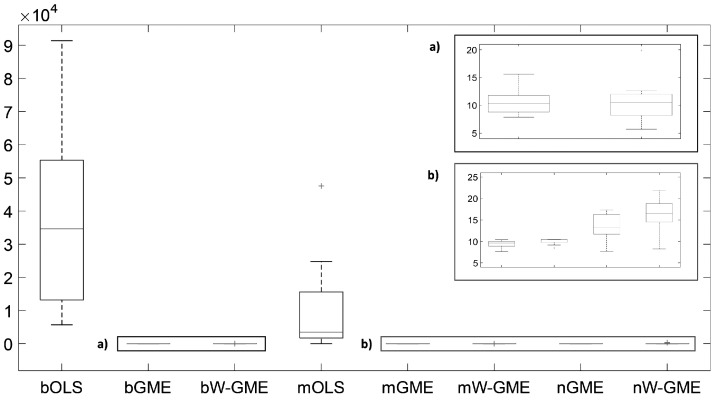
Box plots of the precision errors. Scenario: e∼N(0,1), cond(X) = 20,000, $z_{k}=\left[-200,200\right]$, G = 20, Obs = 100. Note that the y-axis scale of the main plot is of order $10^{4}$. (**a**,**b**) are zoomed-in images of the highlighted box plots.

When comparing the same scenario of normal errors, but with a higher condition number and an increase in the number of groups, in the number of observations, and in the amplitude of the support for the errors (cond(X) =20,000, $z_{k}=\left[-200,200\right]$, G = 20 and Obs = 100), in Table 4, the estimates of the methods previously discussed (namely, bGME, bW-GME, mGME, mW-GME, and nGME) show a low precision error, but only mGME and mW-GME present estimates with less variation compared to the other procedures, as observed in Figure 3. Additionally, the same is observed in Figure 4, where a reduced variance corresponding to these methods in relation to precision error is noted. Thus, the methods highlighted in these scenarios are mGME and mW-GME, with the lowest values of $\left|\right|\hat{\beta }-\beta \left|\right|$ and associated variances, indicating more stable estimates.

**Figure 5:**
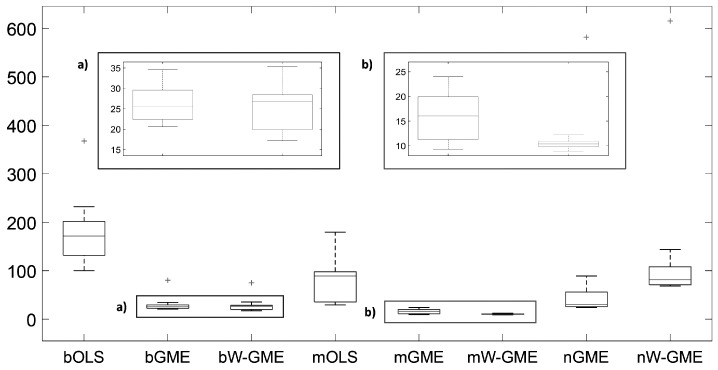
Box plots of the precision errors. Scenario: e∼t(3), cond(X) = 10, $z_{k}=\left[-200,200\right]$, G = 10, Obs = 50. (**a**,**b**) are zoomed-in images of the highlighted box plots.

In the scenario e∼t(3), with a lower condition number, G, and Obs, but $z_{k}=\left[-200,200\right]$, in Table 5, the standout procedure is the magging aggregation methodology using the W-GME estimator due to the reduced $\left|\right|\hat{\beta }-\beta \left|\right|$ and associated variances (see Figure 5). As previously discussed, in this scenario, a decrease in prediction performance of bOLS, nGME, and nW-GME is already visible, along with a general increase in $\left|\right|\hat{y}-y\left|\right|$ in the remaining methods, compared to the scenarios with e∼N(0,1).

**Figure 6:**
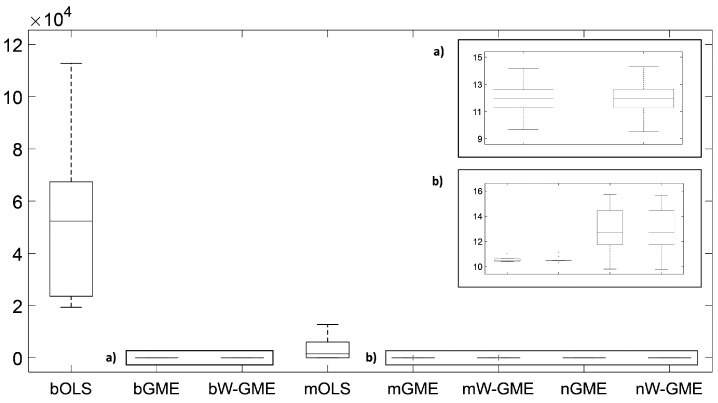
Box plots of the precision errors. Scenario: e∼t(3), cond(X) = 20,000, $z_{k}$=[−10,10], G =20, Obs =100. Note that the y-axis scale of the main plot is of order $10^{4}$. (**a**,**b**) are zoomed-in images of the highlighted box plots.

With the reduction in the amplitude of the support to $z_{k}=\left[-10,10\right]$, and the increase in the condition number, in G, and in Obs, the aggregation methods with estimators based on the ME principle regain prominence, as can be seen from Table 5, both in terms of precision and reduced variance. In particular, the magging method exhibits the best results in terms of $\left|\right|\hat{\beta }-\beta \left|\right|$ and reduced variation in the estimates (see Figure 6). Another evident characteristic is the practically constant prediction error value corresponding to all methods (although higher than in the e∼N(0,1) scenarios).

**Figure 7:**
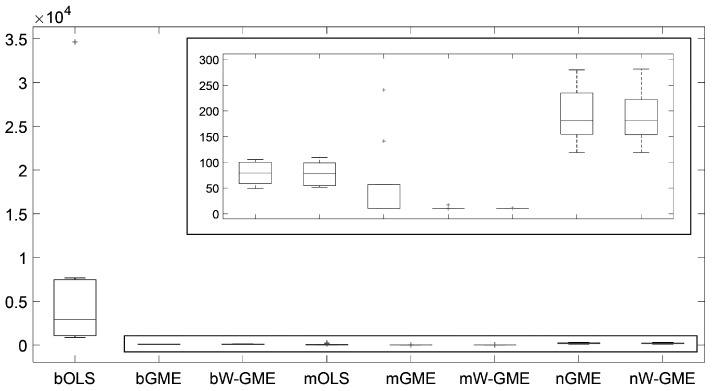
Boxplots of the precision errors. Scenario: e∼C(0,2), cond(X) = 10, $z_{k}=\left[-200,200\right]$, G = 20, Obs = 50. Note that the y-axis scale of the main plot is of order $10^{4}$.

Now, analyzing the situation of e∼C(0,2), with values of G = 20 and Obs = 50 and $z_{k}=\left[-200,200\right]$, the estimates with lower precision error and low variance values come from mW-GME, as can be confirmed in Table 6, further supported by the analysis of Figure 7. However, despite showing low variance, the estimates are excessively centered around zero. Due to the more pronounced presence of noise in this scenario, the standard deviation of the random disturbances, estimated by the standard deviation of the noisy observations, was eventually very high, which may have resulted in obtaining wider error supports. Additionally, the supports for the parameters also have a large amplitude, increasing the impact of the data.

**Figure 8:**
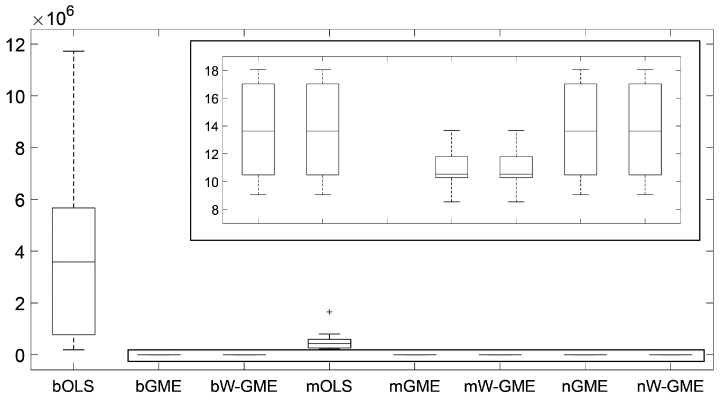
Boxplots of the precision errors. Scenario: e∼C(0,2), cond(X) = 20,000, $z_{k}=\left[-10,10\right]$, G = 10, Obs = 100. Note that the y-axis scale of the main plot is of order $10^{6}$.

To present a comparison with the previous scenario, consider now the situation where $z_{k}=\left[-10,10\right]$, G decreases to 10, and Obs increases to 100, as described in Table 6. Again, the methodologies mGME and mW-GME show good performance, with reduced associated variances and corresponding precision errors with lower variation values, as confirmed in Figure 8.

**Figure 9:**
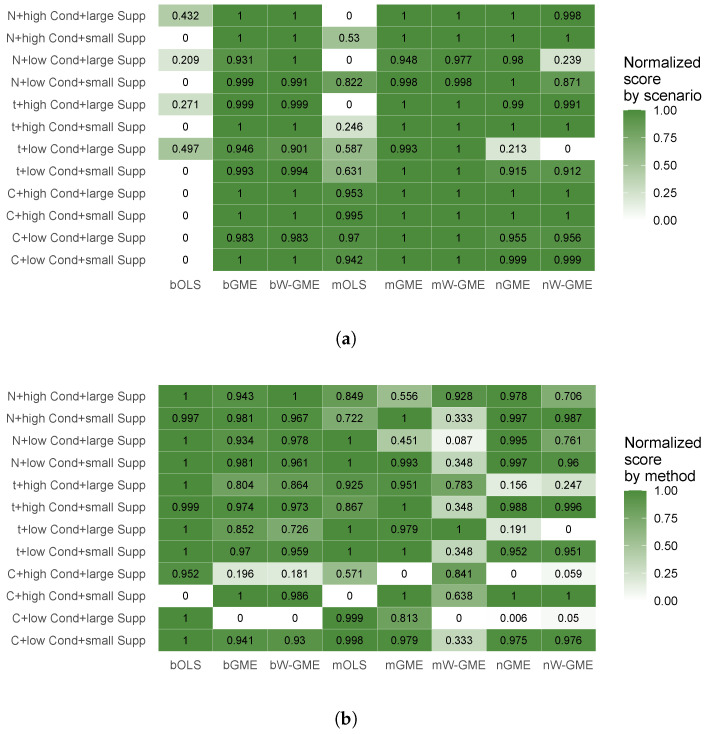
Heatmap of the normalized performance score (scaled from 0 to 1) based on precision error 2, under the condition of the smallest number of groups and of the number of observations per group (G = 10, Obs = 50): (**a**) Precision errors were normalized independently across scenarios. The score allows comparative assessment of methods within each scenario (row), but not across scenarios. (**b**) Precision errors were normalized independently across methods, and enables comparison of scenarios within each method (column). Each simulated scenario is identified on the horizontal axis by the following: error distribution—N (Normal), t (t-Student) or C (Cauchy); collinearity strength—low (cond(X) = 10) or high (cond(X) = 20,000); and range of parameter supports—small ($z_{k}=\left[-10,10\right]$) or large ($z_{k}=\left[-200,200\right]$).

To highlight how different simulated conditions affect performance and to summarize the most relevant relationships, two heatmaps were created. For illustrative purposes, the simulated condition with 10 groups and 50 observations per group (G = 10, Obs = 50) was selected. An inverse min–max transformation was applied to the precision error values (type 2 error was chosen as it represents the precision error of the final aggregated estimate), resulting in a normalized performance score, ranging from 0 (worst) to 1 (best). Figure 9 presents normalized performance scores, with normalization applied independently in two complementary ways: by scenario and by aggregation/estimation method. Panel (a) allows a comparative assessment of methods within each scenario, but not across scenarios. Conversely, in panel (b), the scores enable comparison of scenarios within each method, but not across methods.

As a final technical remark, the method designed for determining the parameter γ is a data-driven method, but different from that suggested in the work of [14], which was the least squares cross-validation (LSCV) method. Due to computational complexity, LSCV is not suitable for big data problems, as it implies thecalculation of *N* estimates of β using the W-GME estimator, for each value of the γ parameter to be tested, for each sample of size *N*. A new method was used in this work for determining this parameter, based on the Holdout methodology, which firstly involves dividing the entire dataset into two mutually exclusive subsets (the training set, $TR_{S}$, and the test set, $TE_{S}$). For each value of the γ parameter to be tested, the β is then estimated, in the training set, using the W-GME estimator, and the prediction error is calculated using the test data, as follows:

(24) $$\hat{S}\left(\gamma \right)=\left|\right|y_{TE_{S}}-X_{TE_{S}}{\hat{\beta }}_{TR_{S}}\left(\gamma \right)\left|\right|.$$

This procedure is executed for any set of possible values of γ that one wishes to test, and, in the end, the value of γ that minimizes the prediction error, $\hat{S}\left(\gamma \right)$, is selected. Although this method aims to determine a reasonable estimate for the parameter γ, and not necessarily the best estimate, it is believed to be sufficient to obtain adequate results, with a much lower computational cost than with LSCV. Using this approach, all values in the range [0,1] with a spacing of 0.01 were tested for the parameter γ.

It was possible to observe the absence of any kind of pattern in the analyzed scenarios, indicating that the weighting parameter γ was being selected based on the data. As in the aggregation context, it is necessary to calculate a parameter γ for each group, it was possible to observe the value of γ adjusting to the data subsets according to the implemented method (it is not possible to present a parameter γ value for each scenario. In the aggregation context, an estimate of the parameter is calculated per group, using the W-GME estimator. Thus, the value of the parameter γ refers to each set of observations (group). Graphical representations available upon request from the authors). As noted earlier, the combination of the magging aggregation procedure with the estimation through W-GME, with this new method of selecting the parameter γ, results in the best outcomes in terms of precision error and low associated variances, among all the aggregation procedures considered with the different estimators discussed. From this, it can be concluded that the new method, although simple, works effectively in determining a reasonable value for the parameter γ, allowing the testing of a variety of values, as many as desired.

## 5. Conclusions

The effectiveness of aggregation methods in solving big data problems is, indeed, remarkable. The parameters of ill-posed linear regression models (particularly, ill-conditioned) in a big data context can be estimated stably through the info-metrics approach. The principle of maximum entropy developed by [2,3] is the theoretical basis for solving ill-posed problems, but the info-metrics approach developed by [4,5] allowed its generalization to more complex ill-posed problems, which are very common in various scientific fields.

The objective of this work was to understand which methodologies are most suitable for big data problems in linear regression models affected by collinearity. The first major conclusion was that the performance of aggregation methods critically depends on the estimator used in the groups obtained by sampling to obtain the respective regression coefficient estimates. The most significant differences highlighted by the simulation work were the diversity of estimates obtained by each aggregation procedure (with the respective applied estimators) and their corresponding precision errors. The prediction error shows significant differences only when the distribution associated with the error component is modified. On the other hand, when the supports for the regression coefficients have a smaller range, assuming that the true value of β is contained within these supports, the choice of the number of groups and the number of observations per group is indifferent in the use of the bagging and magging aggregation methods, using the GME and W-GME estimation methods. This is because the results are approximately similar, presenting reduced precision errors and associated variances, indicating stable estimates. To reduce computational load, a low number of groups and observations per group is sufficient to obtain consistent estimates in the previously described scenario. This is undoubtedly another important result of this work.

The excellent performance of the magging aggregation method using the W-GME estimator was perhaps the most relevant discovery in this research work. When there is no prior information about the supports of unknown parameters and the error distribution, the magging aggregation method with the W-GME estimator shows superior performance in all studied scenarios compared to other aggregation procedures with various estimators used. In particular, the W-GME estimator generally provides better results in terms of precision error (when combined with the magging aggregation method), especially when the supports are wider. This result is quite promising, as the GME estimator, which usually performs well with data affected by collinearity, behaves worse in this circumstance of wider supports (as expected). If the W-GME estimator, being one of its extensions, can handle this problem, it becomes a very attractive estimation method.

Beyond the promising results in the simulation study, the proposed strategies for estimation and aggregation have strong potential for application in diverse real-world domains. These include econometrics, genomics, environmental sciences, and machine learning, where data are often noisy and ill-conditioned. The good performance of GME and W-GME estimators under collinearity and different distributional contexts for the errors, combined with an appropriate aggregation procedure, make them attractive for inference purposes under big data scenarios.

Given the small number of groups and observations per group needed to obtain good results, the computational cost is not a relevant factor, although it is important to keep in mind that the GME and W-GME estimators are slightly slower (constrained nonlinear optimization). Of course, if necessary, parallel computing can be easily implemented, making processing times even shorter.

This work also alerts us to the risk of using the OLS estimator, knowing that data scientists frequently use linear models as a simplified view of the reality in big data analysis, and the OLS estimator is routinely used in practice. Under low collinearity scenarios (condition number of 10), it was verified that the traditional OLS estimator with bagging performs well; however, this strategy tends to become very unstable under high collinearity scenarios (condition number of 20,000), where magging with W-GME stands out in terms of higher performance.

It is important to note some limitations and new avenues of research uncovered by this work. Further research needs to include a broader set of aggregation procedures and estimation methods, which could enhance the comparative framework and potentially offer a more comprehensive view of the context-specific advantages or limitations of each technique (estimation and aggregation), which may not be yet completely revealed [18,19,20]. While the design adopted in this work enabled a systematic and controlled comparison of different methodologies under ill-posed linear regression models, future work will aim to extend the simulation study to account for more realistic scenarios, including nonlinear relationships, heteroscedasticity, skewed distributions for the explanatory variables, and other forms of model misspecification, as well as to test the methods’ performance on real-world datasets.

In summary, as previously mentioned, the identification of the excellent performance of the magging aggregation method, suitable for non-homogeneous data circumstances, using the W-GME estimator, which uses weights in the objective function of the optimization problem to seek a better balance between precision and prediction, was a relevant discovery that could contribute to the analysis of large volumes of information, which is an urgent need in many fields of human activities. Although the entropy-based framework of neagging provides an interpretable aggregation mechanism, its behavior may vary depending on group structure, data quality, and the stability of individual estimators. A deeper exploration of these factors will be fundamental to broadening its applicability. We hope that this work contributes not only by benchmarking methods under controlled conditions, but also by encouraging further research into stable and information-driven aggregation techniques that can meet the challenges posed by modern data environments. 

## Figures and Tables

**Table 1:** Summary table of precision errors 1 and 2 for scenarios related to the data matrix with e∼N(0,1). In each scenario, the lowest precision error is highlighted in bold.

cond(*X*)	$z_{k}$	G/Obs	Type of	Bagging			Magging			Neagging	
			Error	OLS	GME	W-GME	OLS	GME	W-GME	GME	W-GME
10	[−10,10]	10/50	1	86.29	10.46	10.73	45.60	10.48	10.48	**10.44**	16.99
			2	29.51	10.45	10.62	13.84	10.48	10.48	**10.44**	12.90
		10/100	1	34.23	10.33	**10.18**	24.85	10.48	10.49	13.75	14.50
			2	15.15	10.25	9.89	13.55	10.48	10.49	9.72	**8.40**
		20/50	1	52.09	10.41	**10.39**	13.69	10.48	10.48	10.41	17.53
			2	14.38	10.41	10.34	**8.89**	10.48	10.48	10.40	12.65
		20/100	1	24.91	10.22	**9.64**	12.40	10.45	10.47	13.92	11.61
			2	9.67	10.20	9.54	8.46	10.45	10.47	10.43	**6.42**
	[−200,200]	10/50	1	116.43	22.75	21.16	54.84	14.69	**11.22**	25.44	70.89
			2	26.87	11.63	**10.17**	31.28	11.26	10.66	10.59	26.23
		10/100	1	39.15	17.31	15.79	25.82	15.76	**10.38**	22.73	40.36
			2	13.84	8.74	**7.49**	14.79	9.95	9.09	11.42	18.20
		20/50	1	68.26	17.31	18.35	19.50	11.44	**10.51**	19.38	99.55
			2	9.74	8.94	8.85	13.83	10.76	10.50	**8.58**	36.65
		20/100	1	29.83	12.85	12.23	13.56	13.11	**10.23**	16.78	28.06
			2	12.21	**6.92**	7.13	8.40	9.07	10.03	8.03	13.27
20,000	[−10,10]	10/50	1	108,807.14	10.45	10.53	53,822.66	10.47	10.49	**10.42**	13.76
			2	43,621.66	10.45	10.45	20,524.79	10.47	10.49	**10.42**	11.11
		10/100	1	41,645.18	10.21	**10.20**	23,219.29	10.39	10.46	13.55	14.92
			2	6285.39	10.14	10.04	1885.42	10.39	10.46	**9.67**	10.48
		20/50	1	134,468.19	10.44	**10.36**	11,333.22	10.48	10.48	10.41	13.03
			2	30,674.01	10.44	10.34	1133.52	10.48	10.48	10.40	**9.95**
		20/100	1	26,088.79	10.21	**9.91**	5521.06	10.47	10.48	13.56	12.73
			2	12,642.34	10.20	9.85	1508.44	10.47	10.48	10.42	**8.04**
	[−200,200]	10/50	1	160,028.52	23.20	21.74	94,346.67	15.84	**10.76**	26.54	68.17
			2	6329.60	11.41	**9.61**	11,141.37	11.11	10.08	11.66	29.92
		10/100	1	46,393.15	16.28	15.70	29,834.98	11.59	**9.95**	21.25	48.95
			2	5738.78	6.59	6.79	20,050.43	**5.72**	8.71	8.66	30.20
		20/50	1	91,114.53	16.68	15.50	8984.21	**10.52**	10.53	21.27	47.21
			2	44,962.43	**8.30**	8.46	8975.99	10.38	10.52	9.47	18.12
		20/100	1	37,064.06	10.60	10.77	10,508.84	**9.38**	10.02	13.47	44.37
			2	5656.43	**3.69**	3.96	9093.49	9.02	9.92	3.76	30.13

**Table 2:** Summary table of precision errors for scenarios related to the data matrix with e∼t(3). In each scenario, the lowest precision error is highlighted in bold.

cond(*X*)	$z_{k}$	G/Obs	Type of	Bagging			Magging			Neagging	
			Error	OLS	GME	W-GME	OLS	GME	W-GME	GME	W-GME
10	[−10,10]	10/50	1	202.85	11.19	11.17	69.84	**10.47**	10.48	22.73	22.46
			2	44.87	10.71	10.67	23.17	**10.47**	10.48	13.39	13.50
		10/100	1	60.44	12.26	12.22	34.76	**10.37**	10.43	14.65	14.60
			2	42.94	9.86	**9.79**	19.40	10.36	10.35	10.27	10.25
		20/50	1	130.97	11.35	11.31	17.50	**10.48**	**10.48**	22.95	21.78
			2	27.91	11.04	10.97	**10.40**	10.48	10.48	14.44	14.24
		20/100	1	41.95	12.53	12.52	13.14	**10.52**	10.53	13.55	13.53
			2	12.13	11.06	11.07	10.96	**10.51**	10.53	11.22	11.22
	[−200,200]	10/50	1	179.85	31.06	29.84	82.42	16.19	**10.33**	92.55	139.51
			2	43.87	13.68	16.66	37.81	10.50	**10.03**	62.91	77.26
		10/100	1	57.63	87.17	86.21	28.69	**23.30**	26.74	341.40	343.79
			2	**10.15**	36.13	36.89	10.62	10.75	13.46	130.56	130.94
		20/50	1	124.30	21.48	23.15	11.20	10.82	**10.56**	93.96	119.50
			2	28.80	13.22	12.38	10.46	**10.33**	10.51	55.08	43.32
		20/100	1	39.77	51.49	51.45	11.61	**10.39**	12.10	219.09	216.45
			2	12.51	23.44	23.61	10.22	**10.08**	11.24	86.37	83.99
20,000	[−10,10]	10/50	1	193,579.81	11.11	10.91	76,289.47	**10.47**	10.48	21.02	21.30
			2	13,066.07	10.62	**10.31**	9848.51	10.47	10.48	11.06	10.53
		10/100	1	95,677.66	13.74	13.75	36,441.85	**10.50**	10.52	15.29	15.24
			2	24,643.99	11.25	11.26	7486.23	**10.48**	10.51	11.62	11.64
		20/50	1	245,249.84	10.84	10.94	10,325.39	**10.49**	**10.49**	22.79	20.49
			2	41,755.93	10.51	10.60	1449.45	**10.49**	**10.49**	13.25	13.10
		20/100	1	55,489.57	11.89	11.87	3785.99	**10.54**	10.57	12.83	12.81
			2	22,566.09	10.25	10.22	1618.99	10.49	10.51	10.21	**10.20**
	[−200,200]	10/50	1	308,013.72	34.30	33.81	133,724.12	16.12	**10.97**	114.29	147.73
			2	4035.98	14.89	13.11	5534.59	10.54	**10.18**	65.23	60.69
		10/100	1	69,083.72	77.57	76.32	33,072.85	19.06	**15.02**	258.82	259.92
			2	3721.44	34.33	34.23	11,179.60	11.13	**9.81**	113.40	113.81
		20/50	1	135,002.53	22.11	21.25	2172.45	**10.04**	10.44	102.45	127.05
			2	77,503.47	10.79	10.95	1299.89	**9.88**	10.43	51.63	47.99
		20/100	1	45,676.85	55.23	56.39	10,954.65	**10.81**	12.48	240.71	246.11
			2	523.39	28.43	30.17	551.49	**10.10**	10.95	119.62	123.56

**Table 3:** Summary table of precision errors for scenarios related to the data matrix with e∼C(0,2). In each scenario, the lowest precision error is highlighted in bold.

cond(*X*)	$z_{k}$	G/Obs	Type of	Bagging			Magging			Neagging	
			Error	OLS	GME	W-GME	OLS	GME	W-GME	GME	W-GME
10	[−10,10]	10/50	1	7807.18	14.10	14.07	492.17	**10.50**	10.52	16.56	16.55
			2	2280.33	11.45	11.42	143.15	10.50	**10.49**	11.85	11.85
		10/100	1	5561.96	13.84	13.84	267.47	**10.88**	**10.88**	13.84	13.84
			2	3782.26	10.75	10.75	42.82	**9.75**	**9.75**	10.74	10.74
		20/50	1	3246.65	12.18	12.16	46.15	**10.48**	10.49	13.66	13.65
			2	1194.67	10.69	10.69	15.46	**10.48**	10.49	10.73	10.73
		20/100	1	44,481.86	13.10	13.10	39.40	**10.46**	**10.46**	13.10	13.10
			2	42,915.80	11.39	11.39	21.07	**10.37**	**10.37**	11.39	11.39
	[−200,200]	10/50	1	5087.95	131.86	135.29	289.24	16.08	**14.32**	295.25	293.38
			2	1435.85	35.02	35.35	53.45	10.74	**10.72**	74.96	73.93
		10/100	1	95,540.28	225.51	225.53	309.14	**138.16**	138.17	225.06	225.10
			2	93,551.86	84.75	84.81	202.73	**44.36**	**44.36**	84.70	84.89
		20/50	1	6505.53	78.92	78.79	52.46	11.08	**10.52**	194.14	192.51
			2	4648.72	25.86	25.43	34.48	10.74	**10.50**	66.51	65.59
		20/100	1	55,171.34	134.45	134.39	31.16	**19.95**	**19.95**	134.91	134.82
			2	52,819.77	55.50	55.34	11.42	10.82	**10.81**	56.30	56.13
20,000	[−10,10]	10/50	1	18,425,545.05	13.07	13.12	816,608.77	10.47	**10.37**	15.16	15.13
			2	14,091,527.33	**9.97**	**9.97**	73,850.75	10.47	10.28	10.25	10.23
		10/100	1	3,960,459.12	13.56	13.56	544,287.19	**10.94**	**10.94**	13.56	13.56
			2	1,139,209.54	10.86	10.86	140,661.72	**10.38**	**10.38**	10.86	10.86
		20/50	1	498,229,663.96	11.49	11.46	53,777.20	**10.49**	**10.49**	13.26	13.15
			2	493,730,593.87	10.07	10.06	3794.02	10.49	10.49	10.07	**9.97**
		20/100	1	123,549,997.74	12.94	12.94	38,200.42	**10.64**	**10.64**	12.95	12.95
			2	121,549,224.35	11.23	11.23	929.59	**10.52**	**10.52**	11.24	11.24
	[−200,200]	10/50	1	6,984,823.10	116.09	115.91	487,157.74	21.45	**11.75**	265.34	260.49
			2	676,483.39	30.10	30.70	31,685.39	11.91	**10.14**	75.36	73.29
		10/100	1	7,771,232.94	196.80	197.01	417,367.82	**106.57**	107.76	198.21	197.33
			2	3,216,059.63	69.85	68.67	31,103.91	46.85	**45.23**	70.56	69.52
		20/50	1	597,840,024.33	88.56	89.99	54,361.45	**12.96**	13.21	199.86	198.30
			2	591,403,182.85	29.08	29.85	54,354.20	**11.28**	11.53	62.53	61.92
		20/100	1	116,093,776.13	138.41	138.70	134,750.24	**29.62**	29.79	139.90	140.18
			2	108,344,985.84	45.65	45.69	73,639.60	13.08	**13.00**	47.22	47.23

**Table 4:** Prediction errors 1 and 2, and variance of the prediction error 1 for two scenarios related to the data matrix with e∼N(0,1). Scenario A: cond(X) = 10, $z_{k}=\left[-10,10\right]$, G = 10, Obs = 50. Scenario B: cond(X) = 20,000, $z_{k}=\left[-200,200\right]$, G = 20, Obs = 100.

		Bagging			Magging			Neagging	
		OLS	GME	W-GME	OLS	GME	W-GME	GME	W-GME
Scenario A									
	$\left|\right|\hat{y}-y\left|\right|$	184.56	173.14	173.15	176.31	173.14	173.14	173.14	173.91
	$\left|\right|\hat{y}-y{\left|\right|}^{*}$	173.62	173.14	173.14	173.28	173.14	173.14	173.14	173.24
	$s_{\left|\right|\hat{y}-y\left|\right|}^{2}$	50.04	0.00	0.00	6.31	0.00	0.00	0.00	1.13
Scenario B									
	$\left|\right|\hat{y}-y\left|\right|$	173.36	173.19	173.13	173.01	173.02	173.05	173.41	174.48
	$\left|\right|\hat{y}-y{\left|\right|}^{*}$	172.87	172.86	172.85	172.95	173.00	173.05	172.87	172.96
	$s_{\left|\right|\hat{y}-y\left|\right|}^{2}$	0.04	0.03	0.01	0.00	0.00	0.00	0.06	5.94

**Table 5:** Prediction errors 1 and 2, and variance of the prediction error 1 for two scenarios related to the data matrix with e∼t(3). Scenario A: cond(X) = 10, Support = [−200,200], G = 10, Obs = 50. Scenario B: cond(X) = 20,000, Support = [−10,10], G = 20, Obs = 100.

		Bagging			Magging			Neagging	
		OLS	GME	W-GME	OLS	GME	W-GME	GME	W-GME
Scenario A									
	$\left|\right|\hat{y}-y\left|\right|$	320.08	304.14	303.90	306.55	302.57	302.33	341.86	353.92
	$\left|\right|\hat{y}-y{\left|\right|}^{*}$	302.70	302.33	302.42	302.88	302.30	302.32	308.16	311.13
	$s_{\left|\right|\hat{y}-y\left|\right|}^{2}$	121.46	6.67	5.39	10.31	0.04	0.00	12,041.61	13,560.87
Scenario B									
	$\left|\right|\hat{y}-y\left|\right|$	302.94	302.42	302.42	302.36	302.36	302.36	302.48	302.48
	$\left|\right|\hat{y}-y{\left|\right|}^{*}$	302.34	302.34	302.33	302.34	302.36	302.36	302.33	302.33
	$s_{\left|\right|\hat{y}-y\left|\right|}^{2}$	0.17	0.00	0.01	0.00	0.00	0.00	0.01	0.01

**Table 6:** Prediction errors 1 and 2, and variance of the prediction error 1, for two scenarios related to the data matrix with e∼C(0,2). Scenario A: cond(X) = 10, $z_{k}$=[−200,200], G = 20, Obs = 50. Scenario B: cond(X) = 20,000, $z_{k}=\left[-10,10\right]$, G = 10, Obs = 100.

		Bagging			Magging			Neagging	
		OLS	GME	W-GME	OLS	GME	W-GME	GME	W-GME
Scenario A									
	$\left|\right|\hat{y}-y\left|\right|$	804,318.62	804,271.11	804,271.09	804,271.06	804,271.01	804,271.01	804,271.24	804,271.23
	$\left|\right|\hat{y}-y{\left|\right|}^{*}$	804,294.99	804,271.10	804,271.09	804,271.06	804,271.01	804,271.01	804,271.21	804,271.20
	$s_{\left|\right|\hat{y}-y\left|\right|}^{2}$	10,370.59	0.28	0.28	0.02	0.00	0.00	1.54	1.54
Scenario B									
	$\left|\right|\hat{y}-y\left|\right|$	804,273.47	804,270.97	804,270.97	804,270.72	804,270.95	804,270.95	804,270.97	804,270.97
	$\left|\right|\hat{y}-y{\left|\right|}^{*}$	804,272.27	804,270.97	804,270.97	804,270.70	804,270.95	804,270.95	804,270.97	804,270.97
	$s_{\left|\right|\hat{y}-y\left|\right|}^{2}$	32.92	0.00	0.00	0.20	0.00	0.00	0.00	0.00

## Data Availability

The computational structure defined in the manuscript allows the simulation to be replicated. A general code will soon be made public on CRAN, in a user-friendly format. Additional details are available upon request from the authors.

## Abbreviations

The following abbreviations are used in this manuscript:

GME	Generalized maximum entropy
W-GME	Weighted generalized maximum entropy
OLS	Ordinary least squares
bOLS	Bagging aggregation procedure with OLS coefficient estimates
bGME	Bagging procedure with GME estimates
bW-GME	Bagging procedure with W-GME estimates
mOLS	Magging procedure with OLS estimates
mGME	Magging procedure with GME estimates
mW-GME	Magging procedure with W-GME estimates
nGME	Neagging procedure with GME estimates
nW-GME	Neagging procedure with W-GME estimates
G	Number of groups
Obs	Number of observations per group

## Appendix A

To illustrate the algebraic structure of the GME estimator, and considering the same supports used in Section 3, suppose a simple linear regression model, with only ten observations, constructed with an explanatory variable following a U(5,10) and errors following a N(0,1). The coefficients are $\beta_{0}=0.5$ and $\beta_{1}=2.5$, and the response is obtained accordingly to (5). The values of the explanatory and the response variables are rounded to one decimal.

Considering the support spaces as [−200,200] for all the parameters (with M=5) and [−11,11] for all the errors (with J=3) using the three-sigma rule, the GME estimator is given by (10), subject to the model constraints in (11),

$\left[\begin{array}{c} 14.8\\ 17.5\\ 22.2\\ 16.4\\ 14.9\\ 23.1\\ 17.1\\ 18.3\\ 13.1\\ 13.3 \end{array}\right]=\left[\begin{array}{cc} 1 & 5.7\\ 1 & 7.2\\ 1 & 8.1\\ 1 & 6.5\\ 1 & 5.8\\ 1 & 8.5\\ 1 & 6.7\\ 1 & 7.2\\ 1 & 5.3\\ 1 & 5.5 \end{array}\right]\times \left[\begin{array}{cccccccccc} -200 & -100 & 0 & 100 & 200 & 0 & 0 & 0 & 0 & 0\\ 0 & 0 & 0 & 0 & 0 & -200 & -100 & 0 & 100 & 200 \end{array}\right]\times$

$$\;\times \left[\begin{array}{c} p_{1,1}\\ p_{1,2}\\ p_{1,3}\\ p_{1,4}\\ p_{1,5}\\ p_{2,1}\\ p_{2,2}\\ p_{2,3}\\ p_{2,4}\\ p_{2,5} \end{array}\right]+\left[\begin{array}{cccccccccc} -11 & 0 & 11 & 0 & 0 & 0 & \ldots & 0 & 0 & 0\\ 0 & 0 & 0 & -11 & 0 & 11 & \ldots & 0 & 0 & 0\\ \; & \; & & \; & & \; & \ddots & \; & & \\ 0 & 0 & 0 & 0 & 0 & 0 & \ldots & -11 & 0 & 11 \end{array}\right]\times \left[\begin{array}{c} w_{1,1}\\ w_{1,2}\\ w_{1,3}\\ w_{2,1}\\ w_{2,2}\\ w_{2,3}\\ \vdots \\ w_{10,1}\\ w_{10,2}\\ w_{10,3} \end{array}\right],$$

the additivity constraints for p in (12),

$$\left[\begin{array}{c} 1\\ 1 \end{array}\right]=\left[\begin{array}{cccccccccc} 1 & 1 & 1 & 1 & 1 & 0 & 0 & 0 & 0 & 0\\ 0 & 0 & 0 & 0 & 0 & 1 & 1 & 1 & 1 & 1 \end{array}\right]\times \left[\begin{array}{c} p_{1,1}\\ p_{1,2}\\ p_{1,3}\\ p_{1,4}\\ p_{1,5}\\ p_{2,1}\\ p_{2,2}\\ p_{2,3}\\ p_{2,4}\\ p_{2,5} \end{array}\right],$$

and the additivity constraints for w in (12),

$$\left[\begin{array}{c} 1\\ 1\\ 1\\ 1\\ 1\\ 1\\ 1\\ 1\\ 1\\ 1 \end{array}\right]=\left[\begin{array}{cccccccccc} 1 & 1 & 1 & 0 & 0 & 0 & \ldots & 0 & 0 & 0\\ 0 & 0 & 0 & 1 & 1 & 1 & \ldots & 0 & 0 & 0\\ \; & \; & & \; & & \; & \ddots & \; & & \\ 0 & 0 & 0 & 0 & 0 & 0 & \ldots & 1 & 1 & 1 \end{array}\right]\times \left[\begin{array}{c} w_{1,1}\\ w_{1,2}\\ w_{1,3}\\ w_{2,1}\\ w_{2,2}\\ w_{2,3}\\ \vdots \\ w_{10,1}\\ w_{10,2}\\ w_{10,3} \end{array}\right].$$

Considering the support spaces as [−10,10] for all the parameters (with M=5), only the model constraints in (11) need to be adapted as

$\left[\begin{array}{c} 14.8\\ 17.5\\ 22.2\\ 16.4\\ 14.9\\ 23.1\\ 17.1\\ 18.3\\ 13.1\\ 13.3 \end{array}\right]=\left[\begin{array}{cc} 1 & 5.7\\ 1 & 7.2\\ 1 & 8.1\\ 1 & 6.5\\ 1 & 5.8\\ 1 & 8.5\\ 1 & 6.7\\ 1 & 7.2\\ 1 & 5.3\\ 1 & 5.5 \end{array}\right]\times \left[\begin{array}{cccccccccc} -10 & -5 & 0 & 5 & 10 & 0 & 0 & 0 & 0 & 0\\ 0 & 0 & 0 & 0 & 0 & -10 & -5 & 0 & 5 & 10 \end{array}\right]\times \left[\begin{array}{c} p_{1,1}\\ p_{1,2}\\ p_{1,3}\\ p_{1,4}\\ p_{1,5}\\ p_{2,1}\\ p_{2,2}\\ p_{2,3}\\ p_{2,4}\\ p_{2,5} \end{array}\right]+$

$$\;+\left[\begin{array}{cccccccccc} -11 & 0 & 11 & 0 & 0 & 0 & \ldots & 0 & 0 & 0\\ 0 & 0 & 0 & -11 & 0 & 11 & \ldots & 0 & 0 & 0\\ \; & \; & & \; & & \; & \ddots & \; & & \\ 0 & 0 & 0 & 0 & 0 & 0 & \ldots & -11 & 0 & 11 \end{array}\right]\times \left[\begin{array}{c} w_{1,1}\\ w_{1,2}\\ w_{1,3}\\ w_{2,1}\\ w_{2,2}\\ w_{2,3}\\ \vdots \\ w_{10,1}\\ w_{10,2}\\ w_{10,3} \end{array}\right].$$

Using, for example, a nonlinear programming solver (https://www.mathworks.com/help/optim/ug/fmincon.html (accessed on 23 July 2025)) from MATLAB [17], the results are as follows (the products $Z\hat{p}$ are computed with the maximum available precision in MATLAB):

$\left[\begin{array}{c} {\hat{\beta }}_{0}\\ {\hat{\beta }}_{1} \end{array}\right]=\left[\begin{array}{cccccccccc} -200 & -100 & 0 & 100 & 200 & 0 & 0 & 0 & 0 & 0\\ 0 & 0 & 0 & 0 & 0 & -200 & -100 & 0 & 100 & 200 \end{array}\right]\times \left[\begin{array}{c} 0.2063\ldots \\ 0.2031\ldots \\ 0.1999\ldots \\ 0.1968\ldots \\ 0.1937\ldots \\ 0.1939\ldots \\ 0.1969\ldots \\ 0.1999\ldots \\ 0.2030\ldots \\ 0.2061\ldots \end{array}\right]$

$$\;\approx \left[\begin{array}{c} -3.1417\\ 3.0395 \end{array}\right]$$

and

$\left[\begin{array}{c} {\hat{\beta }}_{0}\\ {\hat{\beta }}_{1} \end{array}\right]=\left[\begin{array}{cccccccccc} -10 & -5 & 0 & 5 & 10 & 0 & 0 & 0 & 0 & 0\\ 0 & 0 & 0 & 0 & 0 & -10 & -5 & 0 & 5 & 10 \end{array}\right]\times \left[\begin{array}{c} 0.2031\ldots \\ 0.2015\ldots \\ 0.1999\ldots \\ 0.1984\ldots \\ 0.1968\ldots \\ 0.1096\ldots \\ 0.1429\ldots \\ 0.1865\ldots \\ 0.2433\ldots \\ 0.3174\ldots \end{array}\right]$

$$\;\approx \left[\begin{array}{c} -0.0786\\ 2.5808 \end{array}\right].$$

It is important to note that the results from OLS, defined in (6),

$$\left[\begin{array}{c} {\hat{\beta }}_{0}\\ {\hat{\beta }}_{1} \end{array}\right]\approx \left[\begin{array}{c} -3.2026\\ 3.0485 \end{array}\right],$$

are similar to those from GME with wider support spaces for the parameters, as expected. However, when prior information exists and the supports are narrower, the precision of the estimates substantially increases. This is the reason to consider two different scenarios in Section 3: one where it is assumed some prior knowledge about the range in which the parameters may be found—support spaces as [−10,10] for all the parameters—and another where the available prior knowledge is considered insufficient—support spaces as [−200,200] for all the parameters.
